# Supplementary figures and images for: Mitotic H3K9ac is controlled by phase-specific activity of HDAC2, HDAC3, and SIRT1
Source: Life Sci Alliance. 2022 Aug 18;5(10):e202201433. doi: 10.26508/lsa.202201433 (PMC9389593; doi:10.26508/lsa.202201433)

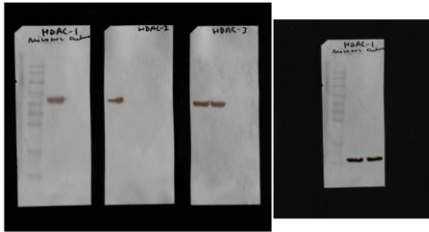

HDAC1 HDAC2 HDAC3 H3

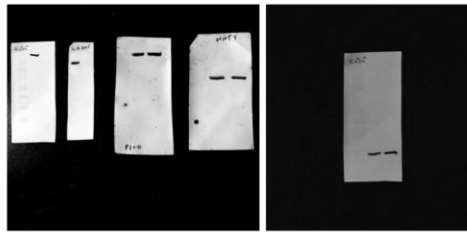

CBP GCN5L2 P300 HAT1 H3

Supplement: Supplementary file 1 [file LSA-2022-01433_SdataF3.pdf]
