# Supplementary material for: Mitotic H3K9ac is controlled by phase-specific activity of HDAC2, HDAC3, and SIRT1
Source: Life Sci Alliance. 2022 Aug 18;5(10):e202201433. doi: 10.26508/lsa.202201433 (PMC9389593; doi:10.26508/lsa.202201433)
Supplement: Supplementary file 2 [file LSA-2022-01433_TableS1.docx]

Table S1 Sequencing depths and ChIP efficiency. Normalization factor to correct for ChIP efficiency and read depth: A factor used to account for differences in ChIP efficiency between samples. This factor was derived by the differences in the chicken promoter average occupancy and read depth.

| <!--Col Count:3-->Sample name | number of reads | Normalization factor to correct for ChIP efficiency and read depth |
| --- | --- | --- |
| Repeat-1 ([Figure 5](#fig5)) |  |  |
| Unsync_H3K9ac_rep1 | 25735442 | 0.629828 |
| Sync_NT_H3K9ac_rep1 | 5761776 | 1.4703905 |
| Sync_RGFP966_H3K9ac_rep1 | 10447729 | 2.747743 |
| Sync_CAY10683_H3K9ac_rep1 | 54414614 | 1.085428 |
| Sync_EX527_H3K9ac_rep1 | 10774458 | 1.222262 |
| Sync_MS275_H3K9ac_rep1 | 70298372 | 2.258310 |
|  |  |  |
| **Repeat-2 (**[**Figure S5**](#figS5)**)** |  |  |
| Sync_NT_H3K9ac_rep2 | 9199420 | 0.554521 |
| Sync_NT_H3K9ac_rep3 | 10635598 | 0.485372 |
| Sync_RGFP966_H3K9ac_rep2 | 11245186 | 2.523053 |
| Sync_CAY10683_H3K9ac_rep2 | 68982790 | 1.480612 |
| Sync_EX527_H3K9ac_rep2 | 26117748 | 0.704151 |
| Sync_MS275_H3K9ac_rep2 | 69855678 | 1.652898 |
